# The dual role of phytoene synthase genes in carotenogenesis in carrot roots and leaves

**DOI:** 10.1007/s11032-014-0163-7

**Published:** 2014-08-08

**Authors:** Hui Wang, Cheng-Gang Ou, Fei-Yun Zhuang, Zhen-Guo Ma

**Affiliations:** Key Laboratory of Horticultural Crop Biology and Germplasm Innovation, Ministry of Agriculture, Institute of Vegetables and Flowers, Chinese Academy of Agricultural Science, No. 12 Nanda Street, Zhongguan Cun, Haidian District, Beijing, 100081 China

**Keywords:** Carrot, Backcross inbred lines (BILs), Phytoene synthase gene, Carotenoids accumulation, De-etiolation

## Abstract

Carrot (*Daucus carota* L.) is an important food crop and is useful for studying carotenogenesis due to the quantity and diversity of carotenoids in its roots. Phytoene synthase catalyzes the first committed step in the carotenoid biosynthesis pathway, and its overexpression is the main driving force in the orange phenotype. At present, we lack fundamental knowledge of the role of these genes and their effects on carotenoid accumulation in leaves. In the present study, three backcross inbred lines (BC2S4) with different colored roots derived from a cross between the orange inbred line (Af) and related wild species were used to investigate the role of the duplicated *DcPSY* genes in root carotenogenesis. Promoter analysis showed that *DcPSY* genes have diverged substantially in their regulatory sequences after gene duplication. Expression levels of *DcPSY1* and *DcPSY2* were generally positively correlated with carotenoid content during root development. In mature leaves, total carotenoid content was higher than that in the roots, *DcPSY1* expression increased extremely higher than *DcPSY2* expression compared with roots, and *DcPSY1* was more sensitive than *DcPSY2* during leaf de-etiolation under sunlight. These results suggest that *DcPSY1* seems to make an important contribution to carotenoid accumulation in the leaves and is important for photosynthesis and photoprotection, but they are not the determining factors of root color. This expands our understanding of the regulation of carotenoid biosynthesis in carrot.

**Electronic supplementary material:**

The online version of this article (doi:10.1007/s11032-014-0163-7) contains supplementary material, which is available to authorized users.

## Introduction

Carotenoids are a diverse family of red, orange and yellow isoprenoid molecules that serve as accessory pigments in the light-harvesting complex and protect the photosynthetic apparatus against photooxidation (Cazzonelli and Pogson 2010). The plant hormone precursors of abscisic acid (ABA) and strigolactones also belong to this class of molecules (Nambara and Marion-Poll 2005; Umehara et al. 2008; Dun et al. 2009). Carotenoids are indispensable nutrients in the diets of mammals, and α- and β-carotene are particularly important as a source of antioxidants and retinoids, and the precursors of vitamin A (Rao and Rao 2007). Vegetables and fruits are considered to be a good source of bioavailable provitamin A carotenoids (Simon 1997).

Carotenoid biosynthesis is a dynamic process that is localized in plastids in higher plants (Shumskaya et al. 2012). Biosynthetic steps include desaturation, cyclization, hydroxylation and epoxidation (Cazzonelli and Pogson 2010; Ruiz-Solaa and Rodríguez-Concepción 2012). Phytoene synthase (PSY) catalyzes the condensation of two geranylgeranyl diphosphate (GGPP) molecules into one phytoene molecule and is the committal step that diverts carbon flux away from competing pathways and toward carotenoid biosynthesis (Hirschberg 2001; Rodríguez-Villalón et al. 2009). Overexpression of *PSY* leads to increased accumulation of carotenoids in rice (*Oryza sativa*) and maize (*Zea mays*) endosperm (Ye et al. 2000; Aluru et al. 2008; Zhu et al. 2008), canola (*Brassica napus*) seeds (Shewmaker et al. 1999), tomato (*Solanum lycopersicum*) fruits (Fraser et al. 2002) and potato (*S. tuberosum*) tubers (Diretto et al. 2010).

In *Arabidopsis*, a single *PSY* gene (*AtPSY*) regulates phytoene synthesis in all tissues (Ruiz-Solaa and Rodríguez-Concepción 2012), while many plants contain two or more *PSY* paralogs that have overlapping roles in carotenogenesis in both photosynthetic and non-photosynthetic tissues. In tomato, *SlPSY1* is primarily responsible for carotenoid accumulation in flower and fruit, and *SlPSY2* performs this function in roots and green tissues (Giorio et al. 2008). In rice and maize, endosperm carotenoid accumulation requires the expression of *PSY1*, while carotenogenesis in photosynthetic tissues requires the expression of both *PSY1* and *PSY2* (Gallagher et al. 2004; Li et al. 2008). Drought and salt stress induces carotenogenesis in roots which enhances ABA, and *PSY3* is required for this process (Welsch et al. 2008). The regulation of *PSY* paralogs remains unclear, but allelic variations in *PSY* could explain carotenogenesis modification in different plant tissues. A delay in lycopene and β-carotene accumulation during tomato fruit ripening was caused by an induced point mutation (P_192_L) in *SlPSY1* (Gady et al. 2012). A single nucleotide polymorphism (SNP) resulting in the A_191_D mutation in a highly conserved region of *MePSY2* enhanced provitamin A levels in cassava roots (Welsch et al. 2010).

Carrot (*Daucus carota* L. var. *sativa*, 2*n* = 2*x* = 18) is one of the most important sources of dietary carotenoids, being particularly abundant in α- and β-carotene (Simon 1997). Naturally occurring single-locus mutations affecting carotenoid accumulation in carrot roots have been documented, including dominant alleles *A* (α-carotene accumulation), *Io* (intense orange xylem), *L*_*1*_ and *L*_*2*_ (lycopene accumulation), *O* (orange xylem), *Y*, *Y*_*1*_, and *Y*_*2*_ (control of differential distribution of α- and β-carotene) as well as recessive alleles *y* (yellow xylem) and *rp* (reduced pigmentation) (Umiel and Gabelman 1972; Buishand and Gabelman 1979; Goldman and Breitbach 1996; Simon 2000). The large quantities of diverse carotenoids contribute to the different colors of carrots and are an ideal model for studying carotenoid biosynthesis (Clotault et al. 2008, 2012). A total of 22 putative genes encoding carotenoid biosynthesis enzymes have been mapped in carrot, but none of the root color alleles appear to be located within these genes (Just et al. 2007, 2009; Cavagnaro et al. 2011). Additionally, the high expression of ζ-carotene desaturase (ZDS) and lycopene ε-cyclase (LCYE) might be consistent with the accumulation of lycopene in red cultivars and lutein in yellow cultivars, respectively; however, this hypothesis was not consistent with α- and β-carotene accumulation in orange cultivars (Clotault et al. 2008).

Orange carrots were not widespread until the fifteenth and sixteenth centuries in Europe (Banga 1957; Stolarczyk and Janick, 2011), and recent allelic diversity of SNP data suggests that they arose from selection of yellow cultivars (Banga 1957; Iorizzo et al. 2013). Phytoene synthesis is the limiting step in carotenoid accumulation in carrot roots (Santos et al. 2005). Increased *DcPSYs* expression was observed in orange carrot roots compared with yellow and white carrots (Bowman et al. 2014). Overexpression of the bacterial *Erwinia uredovora* *PSY* gene *crtB* under the control of a root-specific promoter from yam in wild white carrot cultivar Queen Anne’s Lace (QAL) resulted in increased carotenoid content, which confirmed that *PSY* expression is the rate-limiting step in the transition from white-to-yellow carrots (Maass et al. 2009). Therefore, it remains unclear whether the high α- and β-carotene content in orange roots is controlled by carotenoid biosynthesis pathway genes or other factors. To uncover the role in carotenoid biosynthesis, knowledge of *DcPSY* gene structure and functional information concerning the white-to-orange phenotype change would be valuable, as would insight into the regulatory factors that influence *DcPSY* expression. To date, only limited information has been reported concerning the functional role of carotenoids in photosynthesis and photoprotection in carrot leaves (Stange et al. 2008; Arango et al. 2014; Bowman et al. 2014).

In this study, we determined and analyzed the complete *DcPSY1* and *DcPSY2* gene sequences, including the promoter regions. An SNP and an InDel marker were, respectively, found to differentiate *DcPSY1* and *DcPSY2* between the orange inbred line (Af) and related wild species (Ws) and their backcross inbred lines (BILs; BC2S4) with different colored roots. The overlapping roles of the multiple *DcPSY* genes in the regulation of carotenogenesis in roots and leaves were investigated, and their regulation by light during de-etiolation was also studied.

## Materials and methods

### Plant materials

The orange inbred line Af was selected and self-pollinated for five generations using the European variety *D. carota* var. *sativa* ‘Amsterdam forcing.’ The related wild species *D. carota* var. *carota* ‘Songzi’ (Ws) was supplied by the National Mid-term Genebank of Vegetable Genetic Resources, Chinese Academy of Agricultural Sciences, and self-pollinated for three generations. Orange line E2003, yellow line E36101 and pale orange line E02032 were independently selected from the BC1F1 population of (Af × Ws) × Af, backcrossed with Af again, and then self-pollinated for four generations (BC2S4; Fig. S1). Five accession seeds were directly sown in rows at each 5 m^2^ plot with two agronomic repetitions and had about 300 plants after thinning at the Changping station of the Chinese Academy of Agricultural Sciences on the August 8, 2012.

For gene sequence studies, the roots of the five accessions were randomly sampled from eight plants at the 13th leaf stage. For gene expression and carotenoid accumulation in developmental roots and leaves, the roots of the five accessions were mixed sampled from eight plants with three biological replicates from 0800 to 1000 hours a.m. at the 4th (4 weeks), 5th (5 weeks), 7th (7 weeks), 9th (8 weeks), 11th (10 weeks) and 13th (12 weeks) leaf stages. The leaves of Ws and Af at the 5th and 11th leaf stages were also collected from the eight plants. Both carotenoids and RNA were extracted from the same samples. All samples were frozen in liquid nitrogen and stored at −80 °C until needed.

### Cloning of *DcPSY1* and *DcPSY2*

Genomic DNA was extracted from the roots of Ws, Af, E02032, E36101 and E2003 at the 13th leaf stage using a cetyltrimethyl ammonium bromide (CTAB) method modified from Briard et al. (2000). Primers were designed based on the cDNA sequences of *DcPSY1* and *DcPSY2* (Genebank number: DQ192186 and DQ192187) using Primer Premier 5.0 (http://www.premierbiosoft.com/primerdesign/index.html) as follows: *DcPSY1*, forward, 5′-TTTCACATTTTTAACAAC-3′, reverse, 5′-TAGAGTAACATAATCCCT-3′; *DcPSY2*, forward, 5′-GGAAAAAGACAAACCAAA-3′, reverse, 5′-GAGACCATAAGCAACAAG-3′. Amplifications were performed using TranStart FastPfu DNA Polymerase (TransGen Biotech, China) in a MultiGene OptiMax Thermal Cycler (USA). Polymerase chain reaction (PCR) conditions were as follows: 94 °C for 3 min, 35 cycles of 94 °C for 30 s, annealing temperature (45 °C for *DcPSY1*, 50 °C for *DcPSY2*) for 30 s, 72 °C for 5 min and a final extension at 72 °C for 10 min. Amplified products were analyzed by 1 % agarose gel electrophoresis, cloned into pESY-Blunt Zero Cloning vector (TransGen Biotech, China) and transformed into *Escherichia coli* strain JM109 (TransGen Biotech, China). Clones (60 colonies) were picked individually, cultured in 3 ml Luria–Beratni (LB) medium at 37 °C overnight and sequenced at Beijing Genomics Institute, China. Computer analysis of DNA and amino acid sequences was carried out using DNAman software (Lynnonon Biosoft, Canada) and ClustalW (http://www.ebi.ac.uk/Tools/clustalw2/index.html; Thompson et al. 1994).

### Cloning of *DcPSY* promoters using the genomic walking method

Long and accurate (LA) PCR-based genomic walking was performed to clone the *DcPSY* promoters from Ws and Af using the Genomic Walking Kit (TaKaRa, Japan). Gene-specific primers for cloning the promoters were designed as follows: *DcPSY1*, p1SP1, 5′-ACCATAGATTGCCCAAACAG-3′; p1SP2, 5′-TACTCAGCACAAACTTCGCC-3′; *DcPSY2*, p2SP1, 5′-TCTTTTGACCAACGCTGCTT-3′; p2SP2, 5′-TTACCCTTCTCAAGTCTGCCTC-3′. The primary nested PCR products were diluted 1:100 with distilled water for subsequent nested PCR. All manipulations were carried out as described in the manufacturer’s protocol. DNA sequences of the region 1,557 bp upstream of the translational start ATG codon of tomato *SlPSY1* and *SlPSY2* were obtained from the SOL Genomics Network (http://solgenomics.net), and the corresponding *Arabidopsis* sequences for *AtPSY* (At5g17230) were obtained from the P1 clone MKP11 (GeneBank number: AB005238). Maize *ZmPSY1* (FJ971252.1), *ZmPSY2* (AY325302.1) and *ZmPSY3* (DQ372936.1), and rice *OsPSY1* (297606578), *OsPSY2* (297613623) and *OsPSY3* (297610002) sequences were obtained from the NCBI database. Phylogenetic trees were generated using MEGA 5.05 with the neighbor-joining method (Tamura et al. 2011). Putative *cis*-acting elements within the regions 1,557 bp upstream of *SlPSY1*, *SlPSY2*, *OsPSY3*, *DcPSY1* and *DcPSY2* were identified by searching PlantCare (http://bioinformatics.psb.ugent.be/webtools/plantcare/html/; Lescot et al. 2002).

### UPLC analysis of carotenoids in carrot roots and leaves

Carotenoids were extracted from 200 mg lyophilized roots from the five accessions at the 4th, 5th, 7th, 9th, 11th and 13th leaf stages, and from 100 mg lyophilized leaves from Ws and Af at the 5th and 11th leaf stages using 1 ml of 1:1 acetone:petroleum ether containing 0.1 % butylated hydroxytoluene (BHT). Extracts were dissolved in 2 ml of 1:1 acetone:acetonitrile containing 0.1 % BHT. Carotenoids were separated and analyzed by ultra performance liquid chromatography (Waters, USA). A Waters ACQUITY UPLC BEH C18 Carotenoid column (2.1 mm × 100 mm, 1.7 µm; Waters, USA) was used over 15 min with 1:1 acetone:acetonitrile as the mobile phase. The flow rate was 2 ml/min, the eluent contained 0.01 % BHT to prevent the degradation of carotenoids, and subdued light was used throughout for the same reason. β-Carotene and xanthophylls were identified and quantified based on retention time and peak area compared with authentic standards (Sigma-Aldrich, USA). α-Carotene was calculated according to the β-carotene standard at 450 nm. All extractions and analyses were performed in duplicate. The concentration of each carotenoid was expressed as micrograms per gram dried weight (μg/g DW). Total carotenoid content was estimated by summing the concentrations of α- and β-carotene and xanthophylls. Carotenoid composition pattern was analyzed according to α-, β-carotene and xanthophylls content.

### Expression analysis of *DcPSY* in carrot roots and leaves

Total root RNA from the five accessions at the 4th, 5th, 7th, 9th, 11th and 13th leaf stages and total leaf RNA from Ws and Af at the 5th and 11th leaf stages were extracted using the RNAprep Pure Plant Kit (TianGEN, China). Absorbance at 230, 260 and 280 nm was used for RNA detection and concentration determination using a NanoDrop 2000 (Thermo Scientific, USA). RNA integrity was determined by separation on a 1.2 % agarose gel, and purity was assessed by using the ratio of the absorbance at 260/280 nm and 260/230 nm. Reverse transcription reactions from 1 µg of DNAse I-treated total RNA were carried out using the PrimeScript^®^ RT reagent Kit (TaKaRa, Japan) as described in the instruction manual. Primers for real-time quantitative PCR (qPCR) analysis were designed using Primer Premier 5.0 and verified by BLAST software (http://www.ncbi.nlm.nih.gov/tools/primer-last/index.cgi) according to the *DcPSY1* and *DcPSY2* sequences. Five housekeeping genes including *EF1a* (D12709) (Clotault et al. 2008), *actin* (X17526), *tubulin* (X16608), *ribosomal Protein S10* (AF287338) (Zagon et al. 2010) and *ubiquitin* (Fuentes et al. 2012) were determined in the developmental roots and leaves according to crossing threshold (*C*_t_) values and expression stability by NormFinder (http://www.mdl.dk/publicationsNormFinder.htm). *Ubiquitin* with the lowest stability value (0.262) was amplified along with the target genes as an endogenous control to normalize expression between different samples. The primers were as follows: *DcPSY1*, forward, 5′-CTGACACGGTCTCCACATATCC-3′, reverse, 5′-TCCAACTGTTCCAGCAAC GTA-3′; *DcPSY2*, forward, 5′-GGAACACTACTGATGACCCCA-3′, reverse, 5′-AACTCCCACCTATCCAAAGC-3′; *Ubiquitin*, forward, 5′-GCTCGAGGACGGCAGAAC-3′, reverse, 5′-CTTGGGCTTGGTGTAGGTCTTC-3′ (Fuentes et al. 2012).

Real-time PCR was performed using a StepOne™ Real-Time PCR System (Applied Biosystems, USA). Reactions contained 2 μl diluted cDNA (100 ng), 0.8 μl of each primer (400 nm), 12.5 μl SYBR Green Master Mix and 0.4 μl ROX Reference Dye (TaKaRa, Japan) in a final volume of 25 μl. Reaction conditions were as follows: 95 °C for 3 min, 40 cycles of 95 °C for 5 s, 56 °C for 30 s and 72 °C for 30 s. The specificity of each primer pair was validated by running a dissociation curve (a single slope and peak was observed for each primer pair). Baseline range and *C*_t_ values were automatically calculated using StepOne™ Software v. 2.0 (Applied Biosystems, USA). Each sample underwent three biological and three technical repetitions. The fold change in transcript abundance was calculated as 2^−∆∆^ $$^{{C_{\text{t}} }}$$ (Livak and Schmittgen 2001), and the real-time qPCR amplification data were exported into Microsoft Excel 2003. The expression level of each gene in the roots at the 4th leaf stage of Ws was used for calibration. Values are shown as mean ± SD from the replicates of the eight pooled plants.

### Correlation analysis of *DcPSY* transcript levels and carotenoid content

It is important to know whether and when *PSY* transcript levels correlate with carotenoid accumulation, so that timing can be optimized in breeding and transgenic approaches to enhance carotenoid levels (Li et al. 2008). To relate genotype and root development, the correlation between the transcript levels of the *DcPSY* genes and the carotenoid content in the five accessions was evaluated. The SAS 9.0 statistical software package was used to calculate the Pearson correlation analysis (*r*) and test for statistical significance (*p*). Statistically significant correlation was based on a *p* value ≤0.05. The correlation analysis method was modified from Li et al. (2008).

### *DcPSY* expression during light-triggered de-etiolation

Transcript levels of several genes encoding carotenogenic enzymes can be upregulated by light, leading to carotenoid accumulation (Li et al. 2008; Stange et al. 2008; Toledo-Ortiz et al. 2010). Expression levels of *DcPSY1* and *DcPSY2* were quantified to assess their roles in leaf carotenogenesis during de-etiolation under sunlight. Thirty taproots of Ws and Af within 2 cm petioles of the stem apex were grown in the greenhouse at 13–16 °C (night) and 25–28 °C (day) under continuous darkness, respectively. When approximately six leaves had sprouted, plants underwent 8-h light treatment (natural sunlight from 0700 to 1500 hours), 16-h dark treatment (from 1500 to 0700 hours), followed by 8-h light treatment and 8-h darkness treatment. Leaves were sampled every 4 h between 0700 and 2300 hours (three biological replicates), immediately frozen in liquid nitrogen and stored at −80 °C until needed. A bulk of 6–8 individual leaves sampled from three plants for each sampling time was used with three biological replicates. Total RNA extraction, cDNA synthesis and gene expression analysis were carried out as described above.

## Results

### Variation in *DcPSY* genes between the orange inbred line and wild species

**Fig. 1:**
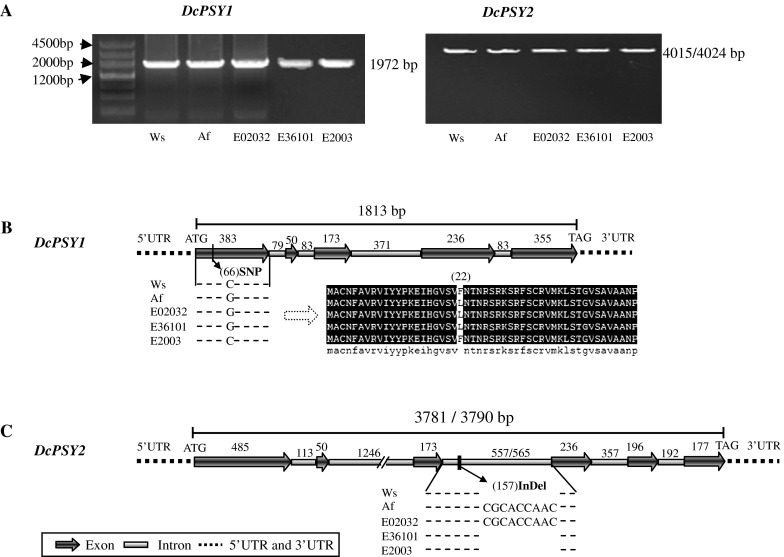
Full-length genomic DNA sequences of *DcPSY1* and *DcPSY2*. **a** Genomic DNA amplification of *DcPSY1* (about 1,972 bp) and *DcPSY2* (about 4,015/4,024 bp) from the roots of Ws, Af, E02032, E36101 and E2003. **b** The genomic DNA sequence of *DcPSY1* from ATG to TAG is 1,813 bp and contains 5 exons and 4 introns. The SNP C_66_G was found in the first exon between Ws and Af, which corresponds to a L_22_F amino acid change*. DcPSY1* of E2003 is from Ws and that of E02032 and E36101 are from Af. **c** The genomic DNA sequence of *DcPSY2* from ATG to TAG is 3,781 and 3,790 bp in Ws and Af, respectively, and contains six exons and five introns. A nine-nucleotide sequence (CGCACCAAC) was found in the third intron between Ws and Af that acts as an InDel marker to differentiate the *DcPSY2* of E36101 and E2003 from that of Ws and that of E02032 from that of Af

In order to understand the variation of *DcPSY1* and *DcPSY2* during breeding, the related wild species *D. carota* var*. carota* Ws and the European orange-type inbred line *D. carota* var. *sativa* Af were selected to clone the total genomic DNA sequence (*DcPSY1*: 1,972 bp; *DcPSY2*: 4,015/4,024 bp) (Fig. 1a). The exon/intron structures of *DcPSY1* and *DcPSY2* were inferred from the alignment of the genomic sequences to the cDNA coding region (Just et al. 2007). The length of the genomic DNA sequence of *DcPSY1* from ATG to TAG was 1,813 bp, which was divided into 5 exons and 4 introns (Fig. 1b). Only an SNP (C_66_G) was present in the first exon of *DcPSY1* between Ws and Af that corresponded to a L_22_F amino acid substitution in the coding sequence. The length of the genomic DNA sequence of *DcPSY2* from ATG to TAG was 3,781/3,790 bp and contained 6 exons and 5 introns (Fig. 1c). The alignment of *DcPSY2* showed only a nine-nucleotide InDel sequence (CGCACCAAC) in the third intron between Ws and Af. Three individual offspring (E02032, E36101 and E2003) were randomly selected from the BIL population. The SNP C_66_G was used to identify the *DcPSY1* of E02032 and E36101 from Af and that of E2003 from Ws (Fig. 1b). The InDel marker was used to identify the *DcPSY2* of E02032 from Af and that of E36101 and E2003 from Ws (Fig. 1c).

### Divergence in the *DcPSY1* and *DcPSY2* promoter structures

**Fig. 2:**
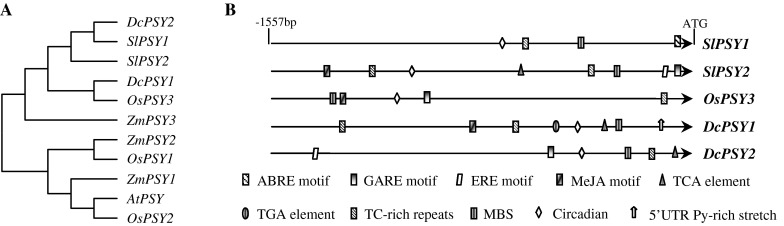
Promoter analysis of *PSY* genes. **a** Phylogenetic tree of *PSY* promoter regions. The promoters of *SlPSY1* and *SlPSY2* were obtained from the SOL Genomics Network (http://solgenomics.net) and that of *AtPSY* (At5g17230) was obtained from the P1 clone MKP11. *ZmPSY1* (FJ971252.1), *ZmPSY2* (AY325302.1), *ZmPSY3* (DQ372936.1), *OsPSY1* (297606578), *OsPSY2* (297613623) and *OsPSY3* (297610002) were obtained from the NCBI database. The phylogenetic tree was generated by MEGA 5.05 using the neighbor-joining method (Tamura et al. 2011). **b** Major putative *cis*-acting elements in the 5′-untranslated regions of *SlPSY1*, *SlPSY2*, *OsPSY3*, *DcPSY1* and *DcPSY2*. Elements were identified using PlantCare and are shown by different character symbols. Only putative *cis*-acting elements associated with defense, hormones, circadian regulation and other particular response elements are shown. Motifs positions are relative and not drawn to *scale*

Promoter studies revealed that *cis*-regulatory motifs are important in mediating transcriptional regulation of *PSY*; phytochrome interaction factors (PIFs) directly bind to the promoter of *AtPSY* and repress expression (Toledo-Ortiz et al. 2010). The putative ABA-response element–coupling element (ABRE–CE) is believed to confer ABA regulation on *OsPSY3* and *ZmPSY3* (Welsch et al. 2008; Li et al. 2009). It was proposed that the regulatory sequences for most plant genes are within 500 bp (or more conservatively, the length of the target gene) upstream from the start codon (Martin et al. 2010). In this study, DNA sequences that were 1,557 bp upstream from the translation start codons of *DcPSY1* and *DcPSY2* in Ws and Af were obtained and analyzed for *cis*-regulatory structures (Fig. 2). This revealed a general lack of similarity for them, but both sequences were the same for Ws and Af indicating that carrot breeding did not affect the promoter region of the *DcPSY*.

Phylogenetic analysis showed that the promoter of *DcPSY1* grouped with *OsPSY3* and that of *DcPSY2* grouped with *SlPSY1* (Fig. 2a). Consistent with light regulation of carotenoid biosynthesis, multiple light-responsive *cis*-elements were identified in the promoter regions (Table S1). Ten light-responsive *cis*-elements (Box 4, GAG motif, BoxII, GATA motif, G-box, I-box, L-box, AAAC motif, ATCT motif and GT1 motif) were found in the *DcPSY1* promoter, and five (Box 4, GAG motif, GA motif, AE motif and BoxI) were present in that of *DcPSY2*. Circadian rhythm-responsive element was found in all analyzed *PSY* promoters (Fig. 2b). Several hormone response elements were identified with differential occurrence. The ABRE ABA-response and MeJA methyl jasmonic acid-response elements were present in the promoters of *SlPSY2*, *OsPSY3* and *DcPSY1*, but absent in that of *DcPSY2*. The GARE GA-response and ERE ethylene-response elements were present in the promoters of *SlPSY2* and *DcPSY2*. The TGA auxin-response element has been shown to interact with carotenoid-derived strigolactone hormones that regulate shoot branching (Hayward et al. 2009), and this element was only found in the *DcPSY1* promoter. The TCA salicylic acid-response element was present in the promoter of *DcPSY1* and *DcPSY2*. Some defense/stress-responsiveness elements were found in the promoters, including the MBS MYB-binding site, which is involved in drought inducibility (Yamaguchi-Shinozaki and Shinozaki 1993), and TC-rich repeats were found in the promoters of *DcPSY1* and *DcPSY2*. Notably, a 5′ UTR Py-rich stretch was only present in the *DcPSY1* promoter, and this *cis*-acting element upregulated transcription in the absence of other upstream *cis*-elements in *Lycopersicon esculentum* (Daraselia et al. 1996).

### Correlation of carotenoid accumulation and *DcPSY* expression in roots

**Fig. 3:**
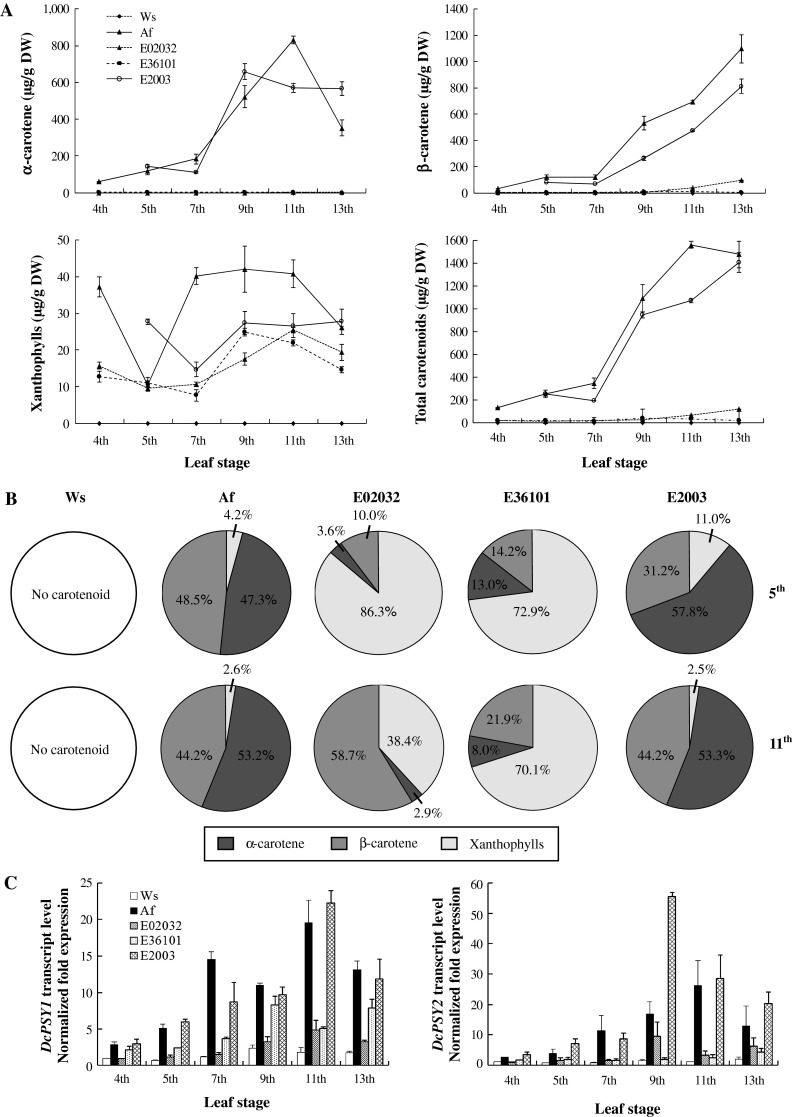
Relationship of carotenoid content and *DcPSY* gene transcription during carrot root development. **a** Carotenoid contents were determined by quantitative UPLC. Total carotenoids were calculated by adding α- and β-carotene and xanthophylls. Results are the means of two extraction replicates ± SD. **b** A comparison of root carotenoid composition patterns among the five accessions was shown at the 5th and 11th leave stage. **c** *DcPSY1* and *DcPSY2* transcription levels were determined by real-time qPCR. The values are normalized against *ubiquitin* transcripts and compared against the expression levels in Ws roots at the 4th leaf stage. Values are the means of three real-time qPCR replicates ±SD

Control of carotenogenic gene transcription is the primary mechanism by which carotenoid biosynthesis and accumulation are regulated in plants (Kato et al. 2004; Qin et al. 2011). The role of transcriptional regulation of *DcPSY* genes in carotenoid accumulation in carrot roots remains uncertain. From the 4th to the 13th leaf stages, carotenoid levels in the roots of the five accessions were determined by UPLC. In Ws roots, carotenoid levels remained below the detection threshold during the six leaf stages (Fig. 3a). For α-carotene, the content in Af roots peaked at 831 µg/g DW at the 11th leaf stage before dropping, while in E2003 roots, this peaked at 658 µg/g DW at the 9th leaf stage before decreasing. A small amount of α-carotene (<5 µg/g) was detected in E02032 and E36101 roots. β-Carotene accumulated slowly in Af, E02032 and E2003 up to the 7th leaf stage, and peaked at the 13th leaf stage at 1,098, 811 and 95 µg/g DW, respectively. E36101 contained a small amount of β-carotene (9 µg/g DW) at the 9th leaf stage. For xanthophylls, accumulation was undulating and peaked at 26 µg/g DW in Af during the 13th leaf stage. Total carotenoid accumulation peaked at 1,563 µg/g DW at the 11th leaf stage in Af, and at 1,407 µg/g DW at the 13th leaf stage in E2003. E02032 and E36101 only accumulated levels of 117 and 38 µg/g DW at the 13th and 9th leaf stages, respectively.

In addition, the carotenoid composition pattern changed during prolonged root development and was different with the exception of Ws (Fig. 3b). The pattern of Af was similar with minor proportion of xanthophylls at the 5th and 11th leaf stages: α-carotene were approximately 47.3 and 53.2 % of total carotenoids, and β-carotene were 48.5 and 44.2 %. The pattern of E2003 turned the same as that of Af at the 11th leaf stage: β-carotene increased from 31.2 to 44.2 %, and α-carotene and xanthophylls decreased from 57.8 to 53.3 % and from 11.0 to 2.5 %, respectively. In E02032, the pattern consisted mostly of xanthophylls (86.3 %) at the 5th leaf stage, and β-carotene (58.7 %) at the 11th leaf stage, and only a minor proportion of α-carotene was present during both stages. In E36101, xanthophylls constituted the major proportion 72.9 and 70.1 % at the two stages, respectively, and levels of β-carotene increased 14.2–21.9 %, but α-carotene decreased from 13.0 to 8.0 %.

*DcPSY1* and *DcPSY2* were expressed in all five accessions (Fig. 3c). Both transcripts were present at lower levels in Ws, but expression increased 2.4-fold for *DcPSY1* and 1.4-fold for *DcPSY2* at the 9th leaf stage. In Af, expression levels for both genes peaked around 20-fold and 26-fold above basal levels at the 11th leaf stage. In E2003, *DcPSY1* expression was upregulated 22-fold at the 11th leaf stage, while *DcPSY2* expression peaked at 55-fold above basal levels at the 9th leaf stage. In E02032, *DcPSY1* expression was upregulated fivefold at the 11th leaf stage, while *DcPSY2* expression peaked at ninefold at the 9th leaf stage. In E36101, *DcPSY1* expression was upregulated eightfold at the 11th leaf stage, while *DcPSY2* expression increased slowly during root developments.

The Pearson correlation was used to probe the relationship between *DcPSY1* and *DcPSY2* expression and carotenoid content (Table S2). In the case of genotype, only expression of *DcPSY1* showed significant correlation with α-carotene (*r* = 0.81, *p* = 0.05) in Af and xanthophylls (*r* = 0.89, *p* = 0.02) in E02032. In root development, expression of *DcPSY1* and *DcPSY2* generally correlated positively with carotenoid content. The 7th leaf stages were the most highly correlated as follows: α-carotene (*DcPSY1*: *r* = 0.99, *p* = 0.002; *DcPSY2*: *r* = 0.99, *p* = 0.0008), β-carotene (*DcPSY1*: *r* = 0.99, *p* = 0.002; *DcPSY2*: *r* = 0.99, *p* = 0.002) and total carotenoid (*DcPSY1*: *r* = 0.99, *p* = 0.002; *DcPSY2*: *r* = 0.98, *p* = 0.003), respectively.

### Correlation of carotenoid accumulation and *DcPSY* expression in leaves

**Fig. 4:**
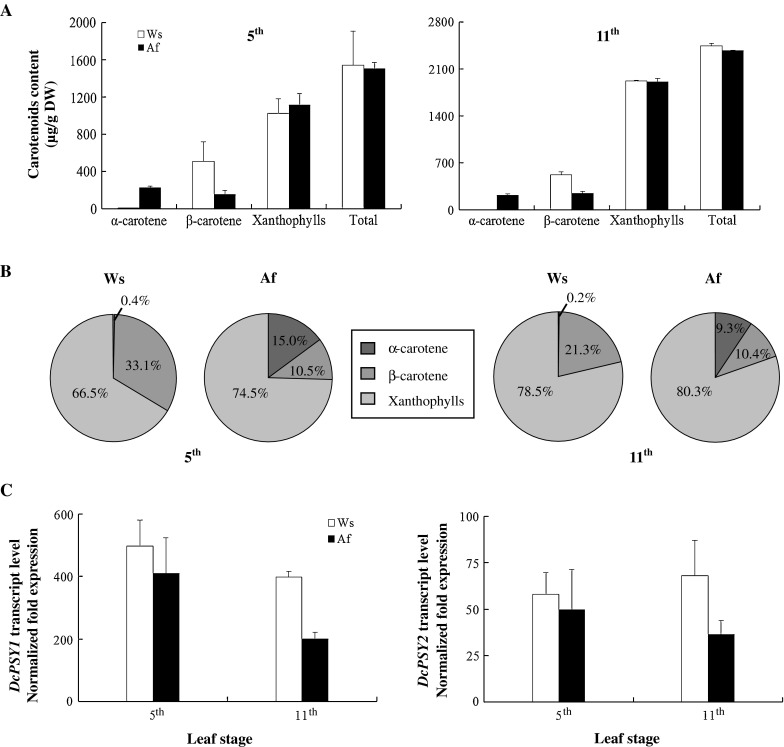
Relationship of carotenoid content and *DcPSY* gene expression in carrot leaves at the 5th and 11th leaf stages. **a** α- and β-Carotene, xanthophylls and total carotenoids were compared between the two stages. **b** Carotenoid composition patterns are shown for Ws and Af leaves. **c** Relative expression levels of *DcPSY1* and *DcPSY2* were compared between Ws and Af leaves. Expression levels are normalized against *ubiquitin* transcripts and compared with expression levels in Ws roots at the 4th leaf stage. Values are the means of three real-time qPCR replicates ±SD

Carotenoids are abundant in leaves where they play an essential role in photosynthesis and photoprotection. The 5th and 11th leaf stages were selected to examine carotenoid content and *DcPSY* expression patterns in Ws and Af. The accumulation of α-carotene was significantly different in the two cultivars (Fig. 4a). Levels reached only 7 and 5 µg/g DW in Ws, but were much higher (226 and 223 µg/g DW) in Af at the two stages. The β-carotene content of Ws (509 and 521 µg/g DW) was higher than that of Af (158 and 248 µg/g DW). Xanthophylls and total carotenoid content were similar for Ws and Af leaves; Ws had 1,023 and 1,923 µg/g DW xanthophylls and 1,538 and 2,449 µg/g DW total carotenoid at the two stages, whereas Af had 1,118 and 1,911 µg/g DW xanthophylls and 1,502 and 2,382 µg/g DW total carotenoids, respectively. The composition pattern also showed very different, especially about 0.2 % α-carotene in Ws but 9.3 % in Af at the 11th leaf stage (Fig. 4b).

Totally, the expression level of *DcPSY1* and *DcPSY2* was higher in Ws, especially at the 11th leaf stage (Fig. 4c). From the 5th to the 11th leaf stage, expression of *DcPSY1* reduced about 20 % in Ws and 51 % in Af, but that of *DcPSY2* increased about 17 % in Ws and decreased about 27 % in Af, respectively. Compared with expression of *DcPSY1* and *DcPSY2* between the leaves and the roots, *DcPSY1* increased 746 times highly than 88 times of *DcPSY2* in Ws at the 5th leaf stage, and 80 times than 13 times of *DcPSY2* in Af (Figs. 3c, 4c), respectively, which was similar at the 11th leaf stage.

### *DcPSY* expression during leaf de-etiolation under sunlight

**Fig. 5:**
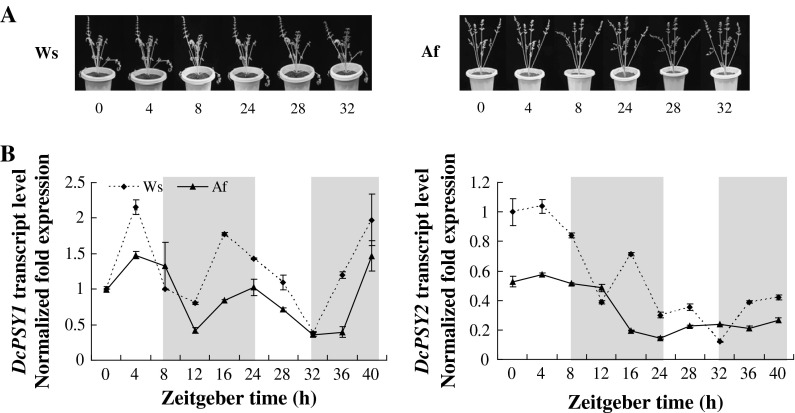
Relative expression levels of *DcPSY1* and *DcPSY2* during de-etiolation under sunlight. **a** Overview of leaf color changes under light treatment for 2 days. Af and Ws are shown on the *left* and *right*. Photographs were taken during the light treatment hours. **b** Relative expression levels of *DcPSY1* and *DcPSY2* under sunlight regulation. Plants were treated with 8-h natural sunlight treatment (from 0700 to 1500 hours), 16-h dark treatment (from 1500 to 0700 hours), followed by 8-h light treatment and 8-h dark treatment. Amplified products are normalized against *ubiquitin* and calibrated against Ws expression levels during the first 4 h of treatment. Values are mean ± SD from three biological replicates

The quantitative and qualitative changes in carotenoid patterns during seedling de-etiolation are associated with concomitant changes in the expression of most genes encoding carotenoid biosynthetic enzymes (Toledo-Ortiz et al. 2010). After 2 days of treatment, the yellow leaves of Ws and Af taproots quickly turned green (Fig. 5a). The expression level of *DcPSY1* increased at the beginning of the 4-h sunlight treatment (2.2-fold for Ws, 1.5-fold for Af) and declined in an undulating manner up to 32 h, at which time the expression was 0.4-fold for Ws and Af (Fig. 5b). Expression had returned to peak level by 40 h. In contrast, expression of *DcPSY2* was relatively unchanged after initial sunlight treatment and declined in an undulating manner. Expression had recovered slightly by 40 h to 0.4-fold for Ws and 0.3-fold for Af. These results suggested that *DcPSY1* is more sensitive than *DcPSY2* during leaf de-etiolation.

## Discussion

Gene duplication events have profound effects on gene function and regulation (Prince and Pickett 2002). Recent data have revealed that gene duplication and subfunctionalization play a central role in recruitment and regulation of carotenoid accumulation in plants (Gallagher et al. 2004; Giorio et al. 2008; Li et al. 2008). PSY catalyzes the first step in the synthesis of carotenoids and is considered to be both the committed and rate-limiting step (Giorio et al. 2008; Li et al. 2008; Qin et al. 2011). Duplicated *PSY* genes are present in carrot and share 59 % sequence identity at the amino acid level, which may allow for a finer, more sophisticated cooperative control of carotenoid biosynthesis and accumulation than occurs in plants with a single *PSY* gene (Clotault et al. 2008). Additionally, *DcPSY1* is more closely related to monocot *PSYs* than it is to other dicot *PSY* genes (Qin et al. 2011). Gene functional studies like the present work offer new opportunities for α- and β-carotene breeding and transgenic fortification.

The BILs investigated in this study were based on an initial cross of Ws (a white wild phenotype) and Af (an orange inbred line; Fig. S1). Their roots have wide color variability that is mainly dependent on the quantities and types of carotenoid present (Nicolle et al. 2004); however, both parents share a similar genetic background. SNP C_66_G in the first exon of *DcPSY1* and the InDel sequence CGCACCAAC in the third intron of *DcPSY2* allowed differentiation of Ws, Af and their offspring (Fig. 1). It is interesting that E02032 with Af *DcPSY1* and *DcPSY2* had pale orange roots and 117 µg/g DW total carotenoid at the 13th leaf stage, while E36101 with Af *DcPSY1* and Ws *DcPSY2* had yellow roots and 38 µg/g DW total carotenoid at the 9th leaf stage, and E2003 with Ws *DcPSY1* and *DcPSY2* had orange roots and 1,407 µg/g DW at the 13th leaf stage (Figs. S1, 3a). However, the carotenoid composition pattern of BILs showed continual changes and some differences compared to Af during prolonged growth (Fig. 3b). These results confirmed that *DcPSYs* do not determine the carotenoid composition pattern and root color directly, which is consistent with the finding that neither *DcPSY1* nor *DcPSY2* are colocated with root color loci (Just et al. 2009; Cavagnaro et al. 2011). However, the expression of *DcPSY1* and *DcPSY2* showed a general positive correlation with carotenoid content in terms of root development experiments (Table S2). Although no carotenoids accumulated in Ws roots, *DcPSY1* and *DcPSY2* were still expressed (Fig. 3c). This indicated that *DcPSYs* play a fundamental role in carotenogenesis of the wild and cultivated roots, but the upregulation that leads to carotenoid accumulation in orange roots may be regulated by other factors. Indeed, the substantial favored β-carotene branch pathway was also found in the transgenic wild QAL plants that expressed the yam *PSY* gene *crtB* under the control of a root-specific promoter. This plant had yellow roots with 10 % β-carotene of total carotenoid in most lines, but α-carotene was absent (Maass et al. 2009). This result is consistent with observations in other engineered plants, such as canola seeds (Shewmaker et al. 1999), rice (Ye et al. 2000), tomato (Fraser et al. 2002) and potato (Diretto et al. 2010).

The dual role of carotenoids in plants probably explains the presence of multiple paralogs that arose from gene duplication and subsequent specialization in different tissues (Li et al. 2009; Qin et al. 2011). Carrots are regarded as the most important source of carotenoids, and much attention has been focused on carrot carotenoid biosynthesis and genetics (Simon 2000; Just et al. 2007, 2009; Cavagnaro et al. 2011; Clotault et al. 2012; Bowman et al. 2014). However, we still lack fundamental knowledge of the role of these genes in controlling carotenoid accumulation in leaves. Compared with roots, total carotenoid content was higher in mature leaves, reaching 2,449 and 2,382 µg/g DW in Ws and Af leaves at the 11th leaf stage (Fig. 4a), respectively, while Af roots only accumulated 1,563 µg/g DW (Fig. 3a). Xanthophylls were the most abundant carotenoids in Ws and Af leaves (Fig. 4b), which are consistent with the critical role of these pigments in photosynthetic light harvesting and photoprotection (Niyogi et al. 1997). Expression analysis revealed that *DcPSY1* transcripts increased extremely higher than that of *DcPSY2* in Ws and Af leaves compared with roots (Figs. 3c, 4c). These results suggest that *DcPSY1* seems to be more important in carotenoid accumulation in photosynthetic tissues.

The mechanisms of carotenoid accumulation were also different between carrot roots and leaves. Interestingly, Af leaves had 30–40 times higher levels of α-carotene than Ws leaves at the two stages, while the content of β-carotene was comparable (Fig. 4a), which is consistent with the results of Arango et al. (2014). This finding differed from the carotenoid composition observed in other plants (Fig. 4b; Britton 1993). A favored α-carotene branch pathway seems to be altered in the leaves during orange carrot breeding (Arango et al. 2014), which is completely contrary to the findings observed in the β-carotene branch pathway found in engineered plants (Shewmaker et al. 1999; Ye et al. 2000; Fraser et al. 2002; Maass et al. 2009; Diretto et al. 2010). This opinion was supported with more wild, purple, yellow and orange carrot accessions. The results showed that wild, yellow and purple carrot leaves have relatively little α-carotene, but this is higher in all orange accessions (data not shown). Moreover, the mechanism of α-carotene accumulation in the leaf appears to be independent in the root (Figs. 3b, 4b; Arango et al. 2014; Bowman et al. 2014).

The upstream regulatory sequences of *DcPSY1* and *DcPSY2* have also diverged following gene duplication; the *DcPSY1* promoter region clusters with *OsPSY3*, while the *DcPSY2* promoter clusters with *SlPSY1* (Fig. 2a). Comparative analysis of the *cis*-regulatory structures in the duplicated *PSY* promoters in tomato, carrot and rice indicates regulation by light, circadian rhythms, abiotic stress and hormones (Fig. 2b; Table S1). Since gibberellins share a common biosynthetic precursor (GGPP) with carotenoids (Sun and Kamiya 1994), ABA is synthesized from the cleavage of apocarotenoids (Nambara and Marion-Poll 2005). Therefore, it is important to understand the significance of the ABRE element in the promoter of *DcPSY1* and the GARE element in the promoter of *DcPSY2* for the regulation of carotenoid accumulation in a metabolic context (Welsch et al. 2008; Li et al. 2009). The auxin TGA-responsive element is only present in the *DcPSY1* promoter, despite interaction between auxin and strigolactones in regulating shoot branching (Hayward et al. 2009).

As well as functioning as accessory pigments in photosynthesis, carotenoids are involved in dissipating excess excitation energy from chlorophyll molecules during de-etiolation or under thermal stress, which is a fundamentally important process for preserving the photosynthetic apparatus (Niyogi et al. 1997; Li et al. 2008; Cazzonelli and Pogson 2010). Light has been shown to repress *DcPSY1* and *DcPSY2* expression in carrot roots (Fuentes et al. 2012), but little information has been reported on the role of sunlight regulation in leaves (Stange et al. 2008). Our system presented an opportunity to address this issue. During leaf de-etiolation, *DcPSY1* expression increased at the start of the 4-h sunlight treatment (Fig. 5b), which could increase production of carotenoids to undergo the transition to photosynthetic development (Rodríguez-Villalón et al. 2009; Toledo-Ortiz et al. 2010). In contrast, *DcPSY2* expression was relatively unaffected following initial sunlight treatment, but decreased markedly following prolonged treatment. After 2 days of treatment, expression of *DcPSY1* recovered to peak levels, but *DcPSY2* expression had only recovered slightly. Both Ws and Af yellow leaves turned to a normal green color (Fig. 5a). These results suggest that *DcPSY1* is more sensitive and plays a major role in assisting photosynthesis and photoprotection during leaf de-etiolation, which is consistent with the greater number of light-responsive elements and stress-response elements in the promoter (Table S1).

In summary, the wide color variability of the BILs used in this study suggests that *DcPSY* genes play a fundamental role in carotenogenesis of wild and cultivated carrot roots, but are not the major factors determining root color. *DcPSY1* and *DcPSY2* expressions were generally positively correlated with carotenoid content in roots, but the factors triggering the increased expression levels in orange roots remain unknown. In mature leaves, total carotenoid content was higher than that in roots, and expression of *DcPSY1* increased extremely higher than that of *DcPSY2* compared with roots, which indicates that *DcPSY1* seems to make a more important contribution to carotenoid accumulation in photosynthetic tissues. The alteration or mutation of the favored α-carotene branch pathway is found in the orange carrot leaves during breeding. The exquisite variation in *DcPSY1* expression during de-etiolation suggests a role in assisting photosynthesis and photoprotection. Further studies to identify and characterize additional carotenoid pathway genes and transcription factors are underway using BIL populations. The results are expected to increase our understanding of the regulation of carotenoid biosynthesis and accumulation in carrot, with particular regard to the mutation(s) that affect α-carotene accumulation in leaves.

## Electronic supplementary material

Below is the link to the electronic supplementary material.

Supplementary material 1 (DOC 820 kb)
