# Supplementary material for: The dual role of phytoene synthase genes in carotenogenesis in carrot roots and leaves
Source: Mol Breed. 2014 Aug 8;34(4):2065–79. doi: 10.1007/s11032-014-0163-7 (PMC4544633; doi:10.1007/s11032-014-0163-7)
Supplement: Supplementary file 1 — Supplementary material 1 (DOC 820 kb) [file 11032_2014_163_MOESM1_ESM.doc]

**Supplementary material**

The following supplementary data can be found at MOLB online.

**Supplementary Fig. S1** Roots of Ws, Af and their backcross inbred lines (BILs) at the 13^th^ leaf stage. Ws (white), Af (orange), E02032 (pale orange), E36101 (yellow) and E2003 (orange)

**Supplementary Table S1** Motifs found in the promoter regions of *DcPSY1* and *DcPSY2*

**Supplementary Table S2** Pearson correlation (*r*/*p*) between *DcPSY1* and *DcPSY2* expression and carotenoid content in roots


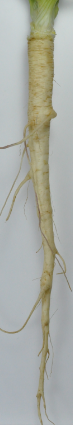

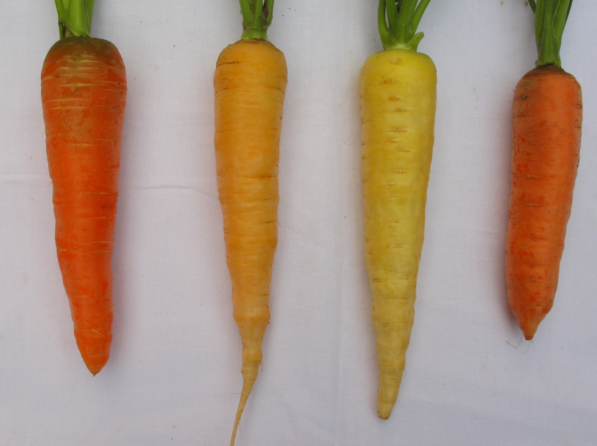


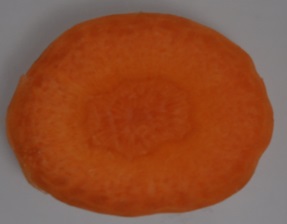

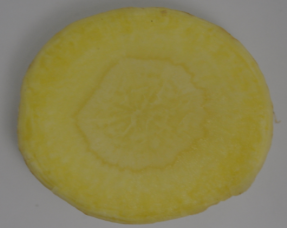

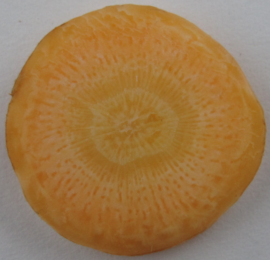

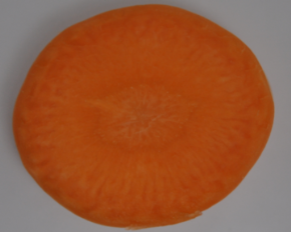

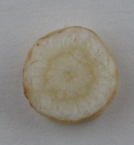


**Ws**

**Af**

**E02032**

**E36101**

**E2003**

**Fig. S1** Roots of Ws, Af and their backcross inbred lines (BILs) at the 13^th^ leaf stage

**Table S1 Motifs found in the promoter regions of *DcPSY1* and *DcPSY2***

| **Motifs** | **Sequences** | **Function** | ***DcPSY1*^a^** | ***DcPSY2*^a^** |
| --- | --- | --- | --- | --- |
| TATA BOX |  | Core promoter element | + | + |
| CAAT box |  | Common *cis*-acting element | + | + |
| Circadian | CAANNNNATC | Circadian regulation | + | + |
| Box 4 | ATTAAT | Light response element | + | + |
| GAG motif | GGAGATG | Light response element | + | + |
| BoxⅡ | ACACGTAGA | Light response element | + | - |
| GATA motif | GATAGGA | Light response element | + | - |
| G box | CACGTA | Light response element | + | - |
| I box | TATTATCTAGA | Light response element | + | - |
| L box | ATCCCACCTAC | Light response element | + | - |
| AAAC motif | CAACAAAAACCT | Light response element | + | - |
| ATCT motif | AATCTGATCG | Light response element | + | - |
| GT1 motif | GGTTAA | Light response element | + | - |
| GA motif | ATAGATAA | Light response element | - | + |
| AE box | AGAAACAT | Light response element | - | + |
| BoxⅠ | TTTCAAA | Light response element | - | + |
| ABRE | TACGTG | ABA responsiveness | + | - |
| TGACG/ CGTCA motif | TGACG/ CGTCA | MeJA response element | + | - |
| GARE motif | TCTGTTG | GA responsiveness | - | + |
| ERE | ATTTCAAA | Ethylene-responsive element | - | + |
| TGA motif | AACGAC | Auxin-responsive element | + | - |
| TCA element | GAGAAGAATA/CCATCTTTTT/CAGAAAAGGA | Salicylic acid responsiveness | + | + |
| ARE | TGGTTT | Anoxia response element | + | + |
| MBS | CAACTG | MYB binding site | + | + |
| TC-rich repeats | GTTTTCTTAC | Defense and stress responsiveness | + | + |
| CCAAT box | CAACGG | MYBHv1 binding site | + | - |
| As-2-box | GATAatGATG | Shoot-specific expression | + | + |
| 5ʹUTR Py-rich stretch | TTTCTTCTCT | Conferring high transcription levels | + | - |
| Skn-1 motif | GTCAT | Endosperm expression regulatory | + | - |
| GCN4 motif | CAAGCCA | Endosperm expression regulatory | + | - |
| Box Ⅲ | atCATTTTCACt | Protein binding site | - | + |
| O2-site | GATGATATGG | Zein metabolism regulation | - | + |
| CAT box | GCCACT | Meristem expression regulatory | - | + |

^a^ + Present; - absent

**Table S2** Pearson correlation (*r*/*p*) between *DcPSY1* and *DcPSY2* expression and carotenoid content

| **Genotype** | | **Ws^a^** | | **Af** | | **E02032** | | **E36101** | | **E2003** | |
| --- | --- | --- | --- | --- | --- | --- | --- | --- | --- | --- | --- |
| ***DcPSY1*** | α-carotene | - | | 0.81 (0.05)* | | 0.67 (0.14) | | 0.33 (0.53) | | 0.56 (0.33) | |
|  | β-carotene | - | | 0.49 (0.32) | | 0.55 (0.26) | | 0.48 (0.34) | | 0.50 (0.39) | |
|  | Xanthophylls | - | | 0.50 (0.31) | | 0.89 (0.02)* | | 0.66 (0.15) | | 0.19 (0.76) | |
|  | Total carotenoids | - | | 0.70 (0.13) | | 0.63 (0.18) | | 0.60 (0.21) | | 0.57 (0.31) | |
| ***DcPSY2*** | α-carotene | - | | 0.70 (0.12) | | 0.42 (0.41) | | -0.51 (0.31) | | 0.83 (0.08) | |
|  | β-carotene | - | | 0.49 (0.32) | | 0.35 (0.50) | | -0.13 (0.81) | | 0.21 (0.74) | |
|  | Xanthophylls | - | | 0.44 (0.39) | | 0.40 (0.43) | | 0.10 (0.85) | | 0.53 (0.36) | |
|  | Total carotenoids | - | | 0.64 (0.17) | | 0.38 (0.46) | | -0.03 (0.96) | | 0.41 (0.49) | |
| **Leaf stage** | | **4^th^** | **5^th^** | | **7^th^** | | **9^th^** | | **11^th^** | | **13^th^** |
| ***DcPSY1*** | α-carotene | 0.84 (0.16) | 0.97 (0.01)* | | 0.99 (0.002)** | | 0.78 (0.12) | | 0.98 (0.02)* | | 0.85 (0.07) |
|  | β-carotene | 0.89 (0.11) | 0.89 (0.04)* | | 0.99 (0.002)** | | 0.79 (0.11) | | 0.98 (0.02)* | | 0.83 (0.08) |
|  | Xanthophylls | 0.81 (0.19) | 0.81 (0.10) | | 0.93 (0.02)* | | 0.91 (0.03)* | | 0.71 (0.18) | | 0.83 (0.09) |
|  | Total carotenoids | 0.84 (0.16) | 0.97 (0.01)* | | 0.99 (0.002)** | | 0.82 (0.09) | | 0.98 (0.02)* | | 0.86 (0.06) |
| ***DcPSY2*** | α-carotene | 0.91 (0.09) | 0.94 (0.02)* | | 0.99 (0.0008)** | | 0.86 (0.06) | | 0.95 (0.01)* | | 0.85 (0.07) |
|  | β-carotene | 0.94 (0.06) | 0.74 (0.15) | | 0.99 (0.002)** | | 0.47 (0.43) | | 0.95 (0.01)* | | 0.84 (0.08) |
|  | Xanthophylls | 0.85 (0.15) | 0.93 (0.02)* | | 0.88 (0.05) | | 0.39 (0.52) | | 0.68 (0.21) | | 0.85 (0.07) |
|  | Total carotenoids | 0.92 (0.08) | 0.89 (0.04)* | | 0.99 (0.002)** | | 0.72 (0.17) | | 0.95 (0.01)* | | 0.91 (0.03)* |

^a^ There were no carotenoids detected in Ws during root development. *, Significant correlation at *p* <0.05; **, significant correlation at *p* <0.01
